# Intrathecal injection of fluorocitric acid inhibits the activation of glial cells causing reduced mirror pain in rats

**DOI:** 10.1186/1471-2253-14-119

**Published:** 2014-12-15

**Authors:** Jing Cao, Zhihua Li, Zhenhua Zhang, Xiuhua Ren, Qingzan Zhao, Jinping Shao, Ming Li, Jiannan Wang, Puchao Huang, Weidong Zang

**Affiliations:** Department of Basic Medical Sciences, Laboratory of Anatomy, Zhengzhou University, Zhengzhou, 450001 Henan Province China

**Keywords:** Mirror-image pain, Satellite glial cells, DL-fluorocitric acid, Nav1.7 protein

## Abstract

**Background:**

Growing evidence has shown that unilateral nerve injury results in pain hypersensitivity in the ipsilateral and contralateral sides respective to the injury site. This phenomenon is known as mirror image pain (MIP). Glial cells have been indicated in the mechanism of MIP; however, it is not clear how glial cells are involved in MIP.

**Methods:**

To observe phenomenon MIP and the following mechanism, 20 adult male Sprague–Dawley rats (weighing 180–220 g) were separated into two groups: Sham Group (n = 10) and left L5 spinal nerve ligated and sectioned (SNL) group (n = 10). Thermal hyperalgesia and mechanical hypersensitivity were measured for both groups to determine if the SNL model had Mirror image of Pain (MIP). Nav1.7 protein expression in DRG was analyzed using immunohistochemistry and western-blotting. And then to observe the effect of fluorocitrate on MIP, 15 rats were separated into three Groups: Sham Group (n = 5); SNL + FC group: intrathecal injection of Fluorocitric acid(FC) 1 nmol/10 *μ*L (n = 5); SNL + NS group: intrathecal injection of 0.9% Normal Saline (n = 5). Behavior testing, immunocytochemistry, and western-blotting using dorsal root ganglion (DRG) from both sides were then conducted.

**Results:**

The results showed pain hypersensitivity in both hind-paws of the SNL animals, Mechanical tests showed the paw withdrawal threshold dropped from 13.30 ± 1.204 g to 2.57 ± 1.963 g at 14 d as will as the ipsilateral paw thermal withdrawal threshold dropped from 16.5 ± 2.236 s to 4.38 ± 2.544 s at 14d. Mechanical tests showed the contralateral paw withdrawal threshold dropped from 14.01 ± 1.412 to 4.2 ± 1.789 g at 7d will the thermal withdrawal threshold dropped from 16.8 ± 2.176 s to 7.6 ± 1.517 s at 7d. Nav1.7 expression increased and glial cells actived in bilateral side DRG after SNL compared with sham group. After intrathecal injection of fluorocitrate, the glial cell in bilatral DRG were inhibited and the pain behavior were reversed in both hindpaws too.

**Conclusions:**

Fluorocitrate can inhibit the activation of glial cells in spinal cord and DRG, and reduce MIP.

## Background

A growing body of evidence indicates that unilateral nerve injury results in bilateral cellular and molecular changes in the nerve structure and pain sensitivity [1, 2]. This phenomenon is known as MIP [3]. To date, the mechanism of MIP is still unclear. Deepak Behera [4] used Manganese-enhanced magnetic resonance imaging (MRI) to show increased manganese uptake in both the injured sciatic nerve and contralateral sciatic nerve in the chronic constriction injury model of neuropathic pain. Although poorly understood, this finding corroborates the *ex vivo* finding of bilateral nociceptive-related molecular changes in the nervous system of unilateral pain models. It may be related to humoral immunity, central sensitization, and/or cortical downstream regulation. Surprisingly, evidence of changes in primary neurons and satellite glial cells (SGCs) in regards to MIP is lacking. Because of their unique location in sensory and autonomic ganglion, SGCs can strongly influence nociceptive sensation [5].

In our preliminary studies we found *SCN9A* abnormality highly expressed in both bilateral spinal ganglion which correlates with the development of MIP. High expression of Nav1.7 protein in the contralateral side may explain the increase in neuronal in the mirror side. *SCN9A* encodes a subunit of the voltage-gated channel Nav1.7, in which a single-gene mutation is closely related to a congenital abnormality in which the sensation of pain is lost [6]. Yang yong [7] reported a gain-of function mutation of *SCN9A* causes erythema acrodynia, a disease of severe episodic pain.

Nav1.7 may be a promising candidate for the cause of MIP, but the exact mechanism of its upregulation and the associated increase in neuronal excitability is still unkown. It is possible that SGCs in the contralateral DRG may play a role in primary neuronal sensitization [8, 9]. SGCs are found in the peripheral nervous system, particularly in DRG. SGCs are the main glial cells in DRG, and they become activated and proliferate after nerve injury or inflammation [10]. SGCs are arranged in a layer, normally around the neurons to form a complete scabbard film. The SGCs also release substances after nerve injury, which can directly affect the neurons that the SCGs surround [11]. Based on the close proximity of the SGCs and their ability to affect primary neurons, we hypothesize that SGC activation in the contralateral DRG following unilateral peripheral nerve injury leads to increased excitability of contralateral DRG neurons and thus, MIP.

To address this hypothesis, a rat MIP model established by nerve distal ligation and section (SNL) was used to identify changes in Nav1.7 expression and SGCs activation. Molecular techniques including RT-PCR, western-blotting, and immunohistochemistry were used to identify changes in the expression of Nav1.7 in DRG. Behavioral tests were also utilized to measure pain hypersensitivity. DL-Fluorocitric acid was used to inhibit SGCs activation, and verify the role of SGCs in Nav1.7 overexpression and pain hypersensitivity.

## Methods

### Animals and surgical procedures

Adult male Sprague–Dawley rats (6-8 W) of clean grade, weighing 180–220 g(n = 25), were provided by the Experimental Animal Center of Henan Province (license No. SYXK2005-0012). The rats were housed with a 12-hour light–dark cycle and free access to food and water. They were kept for 1 week under these conditions before surgery. All procedures were performed in accordance with the Guidance Suggestions for the Care and Use of Laboratory Animals, formulated by the Ministry of Science and Technology of China [12]. To produce persistent neuropathic pain, SNL was performed according to our previous protocols. Briefly, rats were anesthetized with chloral hydrate (300 mg/kg, i.p.). A midline incision was then made at the L3–S1 level, and the dorsal vertebral column from L4 to S1 was exposed. The left L5 spinal nerve was carefully isolated and tightly ligated and sectioned distal to the DRG with 6–0 silk thread. Sham-operated animals were subjected to a similar surgical procedure in which the spinal nerves just be isolated.

### Intrathecal injection

A PE10 polyethylene tube was prepared and used as an injection catheter. The injection catheter was pre-filled with 10 μl of fluorocitrate 1 nmol/10 *μ*L. (Fluorocitrate (FC) was purchased from Sigma-Aldrich Chemical Co. (St. Louis, MO, U.S.A.)) or vehicle (0.9% saline) and 10 μl of saline separated by a small air bubble. Under anesthesia, tissue between two spinous processes of lumbar vertebrae L5 and L6 were seperated, A 21-gauge sterile needle was inserted into ligamentum flavum, and some cerebrospinal fluid overflowed. The PE10 polyethylene tube was inserted into the lumber enlargement and advanced about 3 cm, where its arrival was confirmed by a tail-flick. The PE10 polyethylene tube was fixed to the neck under skin. Intrathecal injection was performed directly into the subarachnoid space of the lumbar enlargement. After surgery, neurologically normal rats were injected with 2% lidocaine (10 μL) through the intrathecal catheter to confirm that the PE10 tubing was in the subarachnoid space. Only those rats showing complete paralysis of both hind limbs and the tail after the administration of lidocaine were used for the subsequent experiments. The FC, or vehicle, was injected and followed by a 0.9% saline flush. At the end of each experiment, the position of the PE10 tubing in the intrathecal space at the lumbar enlargement was visually verified by exposing the lumbar spinal cord.

### Mechanical hypersensitivity

Rats were placed on an elevated mesh grid that completely exposed the middle of the hind paw. Mechanical hypersensitivity was tested using von Frey filaments (Stoelting, Kiel, WI, USA) by experimenters who were blinded to group assignment. Each filament was perpendicularly applied to the mid-plantar surface of the hindpaw. Withdrawal thresholds were determined using sequentially increasing and decreasing stimulus strength “up-and-down” method. The forces of the von Frey filaments were 2, 4, 6, 8, 10, and 15 g. The 2 g stimulus was applied first. If a positive response occurred, the next smaller von Frey hair was used; if a negative response was observed, the next larger von Frey hair was used. The test was ended when (i) a negative response was obtained with the 15 g hair, (ii) four stimuli were applied after the first positive response, or (iii) nine stimuli were applied to one hind paw.

### Thermal sensory testing

Rats were habituated to the thermal testing apparatus (Type PL-200, ChengDu Techenology and market Co. LTD. ChengDu, China) for 30 minutes. Rats were placed in a Plexiglas chamber on a glass plate. A radiant heat was applied by aiming a beam of light through a hole in the light box through the glass plate to the middle of the plantar surface of each hind paw. When the animal lifted its foot, the light beam was turned off. The length of time between the start of the light beam and the foot lift was defined as the paw withdrawal latency. Each trial was repeated five times at 5-min intervals for each side. A cut-off time of 20 s was used to avoid tissue damage to the hind paw.

### Immunohistochemistry

The rats were deeply anesthetized by injection of pentobarbital (60 mg/kg, i.p.) and transcardially perfused with 200 mL of 5 mM sodium phosphate-buffered 0.9% (w/v) saline (PBS, pH 7.3), followed by 500 mL of 4% (w/v) paraformaldehyde in 0.1 M phosphate buffer (PB, pH 7.4). The L5 spinal cord segments and DRGs were harvested and fixed by 4% (w/v) paraformaldehyde. The tissue was embedded in paraffin, and cut into 4-μm thick sections using a vibratome. Tissue sections were dewaxed and washed, and then maintained in 3% H_2_O_2_ for 20 minutes at 37°C, followed by blocking in 10% goat serum for 60 minutes at 37°C. The sections of DRG were incubated in anti-Nav1.7 rabbit IgG (1:500; Chemicon, Temecula, CA, USA) or anti-glial fibrillary acidic protein (GFAP) mouse IgG (1:500; Chemicon, Temecula, CA, USA); the sections of L5 spinal cord were incubated with anti-glial fibrillary acidic protein (GFAP) mouse IgG (1:500; Chemicon, Temecula, CA, USA) at 4°C for 12 hours, washed three times with PBS to remove excess antibodies, incubated in goat anti-rabbit IgG or goat anti-mouse IgG conjugated to biotin (1:100; Biosynthesis Biotechnology, Beijing, China) at 37°C for 60 minutes, washed three times with PBS for 5 minutes each. Antibody binding was visualized with the ABComplex/HRP. Staining was performed with DAB and counterstaining with hemalum. Images were collected using a DMI3000 B Leica microscope (Leica, Wetzlar, Germany).

### Double immunofluorescent-labeling Nav1.7 with GFAP

For Double immunofluorescent-labeling, 7 day post-SNL animals were injected with pentobarbital (30 mg/kg, i.p.) and transcardially perfused with 200 mL of 5 mM sodium phosphate-buffered 0.9% (w/v) saline (PBS, pH 7.3), followed by 500 mL of 4% (w/v) paraformaldehyde in 0.1 M phosphate buffer (PB, pH 7.4). After the perfusion, the DRG was dehydrated by an ethanol gradient, embedded in paraffin, and then sliced at a thickness of 4 μm. After dewaxing with dimethylbenzene and hydration by an ethanol gradient,and antigen heated repair ,these sections were incubated with 3% H_2_O_2_ at 37°C for 20 min, and then were blocked with 2% goat serum in 0.3% Triton X-100 for 1 h at room temperature (RT) and incubated overnight at 4°C with a mixture of anti-Nav1.7 rabbit IgG (1:500; Chemicon, Temecula, CA, USA) and anti-GFAP mouse IgG (1:500; Chemicon, Temecula, CA, USA), then washed three times with PBS to remove excess antibodies, followed by a mixture of the two respective secondary antibodies of goat- anti-rabbit TRITC (1: 1000, Beijing Zhongshan Golden Bridge Biotechnology Co. Beijing, China) and goat-anti-mouse FITC (1:1000, Beijing Zhongshan Golden Bridge Biotechnology Co. Beijing, China). Stained sections were examined with a Nikon (Tokyo, Japan) fluorescence microscope, and images were captured with a CCD Spot camera.

### Western blot analysis

Animals were deeply anesthetized by injection of pentobarbital (60 mg/kg, i.p.) and then rapidly sacrificed. The DRGs and L5 spinal cord segments were dissected on ice according to the termination of the L4 and L5 dorsal roots. The left dorsal part of spinal cord was further split and then homogenized with a hand-held pestle in SDS sample buffer (10 mL/mg tissue) containing a mixture of proteinase and phosphatase inhibitors (Sigma, MO, USA). The protein concentrations were estimated using the bicinchoninic acid (BCA) method. The samples were heated in boiling water for 8 min, loaded onto 10% SDS-polyacrylamide gels, and transferred to polyvinylidene difluoride membranes (PVDF, Immobilon-P, Millipore, Billerica, MA, USA). Membranes were blocked in a 3% no-fat milk solution for 1 hour and probed with the following primary antibodies overnight at 4°C: anti-GFAP mouse IgG (1:5000; Chemicon, Temecula, CA, USA), anti-Nav1.7 rabbit IgG (1:200; Chemicon, Temecula, CA, USA ), and anti-β-actin mouse IgG (1:3000, Sigma). The membranes were rinsed three times (10 minutes each) with Tris-buffered saline with Tween-20 (TBST) between each step, the membranes were then incubated with the following secondary antibodies for 2 hours: HRP-conjugated anti-rabbit IgG (1:5000; Beijing Zhongshan Golden Bridge Biotechnology Co. Beijing China) and HRP-conjugated anti-mouse IgG (1:5000; Beijing Zhongshan Golden Bridge Biotechnology Co. Beijing,China). The membranes were rinsed three times (10 minutes each) with Tris-buffered saline with Tween-20 (TBST) between each step. All reactions were detected by the enhanced chemiluminescence (ECL) detection method (Amersham). The densities of protein blots were analyzed using Labworks Software (Ultra-Violet Products, UK). The densities of target proteins and β-actin immunoreactive bands were quantified with background subtraction. The same size square was drawn around each band to measure the density and the background near that band was subtracted. Target protein levels were normalized against β-actin levels and expressed as relative fold changes compared to the Sham-Veh group.

### Statistical analysis

All data were expressed as mean ± s.e.m. For electrophysiology, those cells which showed a >5% change from the baseline level during drug perfusion were regarded as responding cellsand were usedfor statistical analysis [13]. Differences between groups were compared using a student t-test or a one way ANOVA test. The criterion for statistical significance was P < 0.05.

## Results

### Changes in the behavior of SNL rats

**Figure 1:**
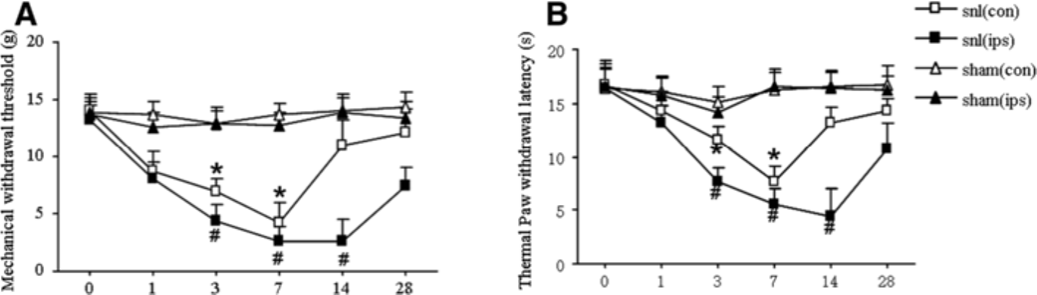
**Behavioral changes after L5 spinal nerve ligation in rats. A**: Mechanical hyperalgesia. after L5 spinal nerve ligation in rats. The threshold of contralateral paw of SNL model were dropped within 14 days after surgery ,the lowest is at 7d. ^#^ *P* < 0.05 vs ipsilateral paw of sham group, *P < 0.05 vs contralateral paw of sham group; **B**: Thermal hyperalgesia after L5 spinal nerve ligation in rats. The threshold of ipsilateral paw of SNL model were dropped within 28 days after surgery ,the lowest is at 14d. The threshold of contralateral paw of SNL model were dropped within 14 days after surgery ,the lowest is at 7d. ^#^ *P* < 0.05 vs ipsilateral paw of sham group, *P < 0.05 vs contralateral paw of sham group; n = 30.

Unilateral SNL rats exhibited noticeable bilateral pain. Compared with the sham group, the ipsilateral paw pain thresholds for thermal and mechanical hyperalgesia were significantly increased at 3–28 d following operation (P < 0.05; Figure 1). Mechanical tests showed the paw withdrawal threshold dropped from 13.30 ± 1.204 g before operation to 2.57 ± 1.963 g at 14 d after operation. The ipsilateral paw thermal withdrawal threshold dropped from 16.5 ± 2.236 s before operation (baseline) to 4.38 ± 2.544 s at 14 d after operation. Compared with the sham group, the contralateral paw pain thresholds for thermal and mechanical hyperalgesia were increased at 3–7 d following operation as well (P < 0.05; Figure 1). Mechanical tests showed the contralateral paw withdrawal threshold dropped to 4.2 ± 1.789 g from 14.01 ± 1.412 at 7 d post operation. The contralateral paw thermal withdrawal threshold dropped to 7.6 ± 1.517 s from 16.8 ± 2.176 s at 7 d post operation. Allodynia remained at the peak level for more than 7 d.

### Nav1.7 expression in the L5 DRG

**Figure 2:**
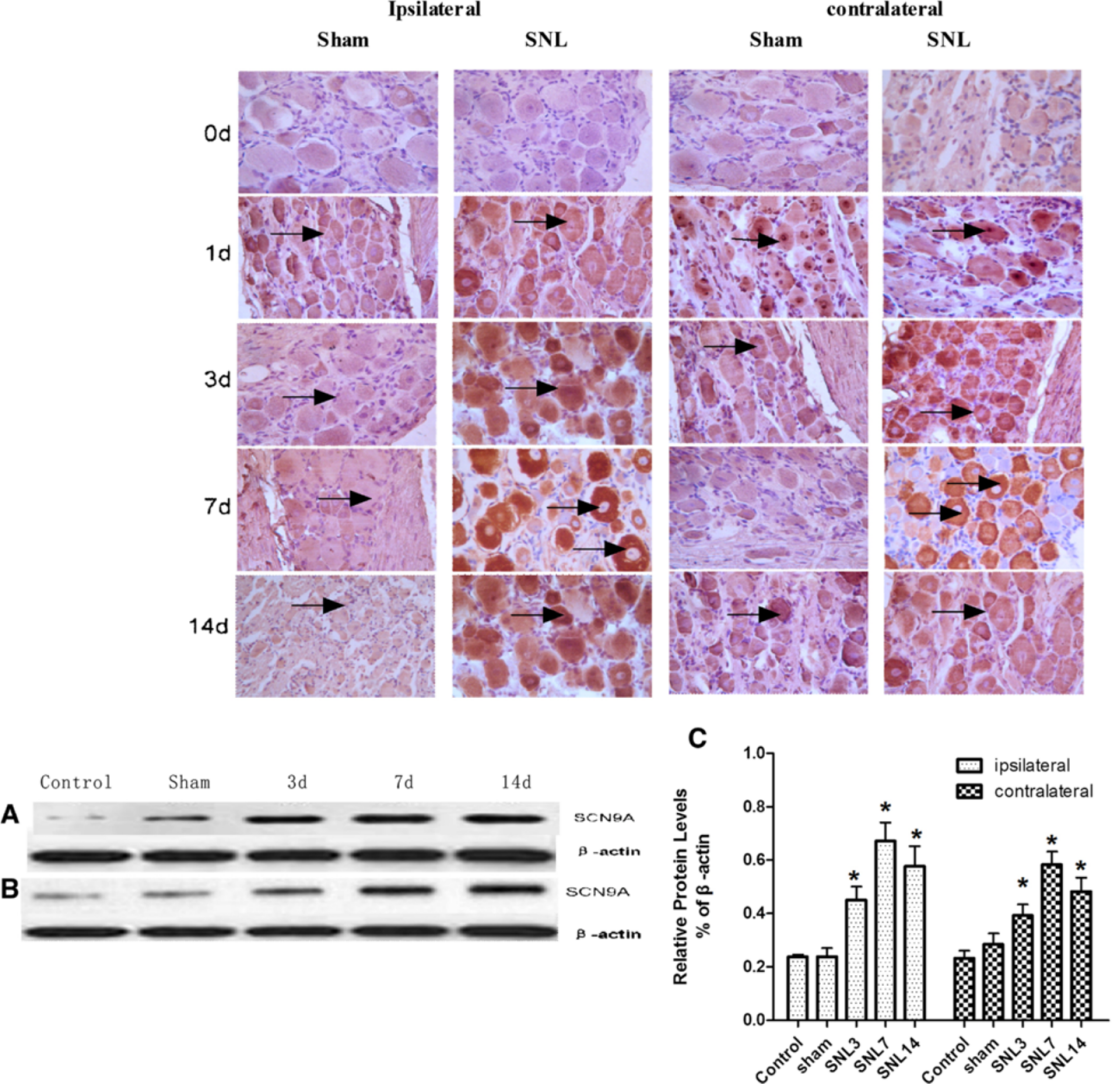
**Expression of Nav1.7 in bilateral ganglion.** The expression of Nav1.7 in bilateral ganglion by Immunohistochemistry **A**: The positive cell was marked by arrow. compared with the sham group, both side ganglion showed the expression of Nav1.7 was increased significantly, especially at 3d, 7d,14d. **B**: The expression of Nav1.7 in bilateral ganglion by western staining. B1:Compared to the Sham group, Westernblot showed the expression of Nav1.7/ SCN9A in ipsilateral ganglion increased significantly, especially at 7d,14d. B2: Compared to the Sham group, Westernblot showed the expression of Nav1.7/ SCN9A in contralateral ganglion increased significantly, especially at 7d. **C**: the graph for statistical *P < 0.05 vs Sham group (n = 3).

Immunohistochemistry experiments revealed Nav1.7-positive neurons in the injured L5 DRG specimens. Compared with the sham groups, immunohistochemistry experiments revealed a significant increase in Nav1.7 protein expression in the injured L5 DRG specimens at day 14 post-SNL. Compared with the sham group, there was also a significant increase in the expression of Nav1.7 in the injured L5 DRG at 1d, 3d, and 7d post-SNL (Figure 2). These results indicate that SNL induced long-lasting Nav1.7 activation in the bilateral DRG. And the Western blot results indicated that the Nav1.7 expression level changed with behavioral alterations in the rat right hind-paw pain model in SNL groups. Compared with the sham group, Western blot showed Nav1.7 protein was significantly increased in the injured DRG of the SNL group, especially at 7 d post-SNL, the ratio to sham group is up to 0.581 ± 0.070 from 0.231 ± 0.041 at 7d (*P < 0.05)

### Activation of satellite cells after SNL

**Figure 3:**
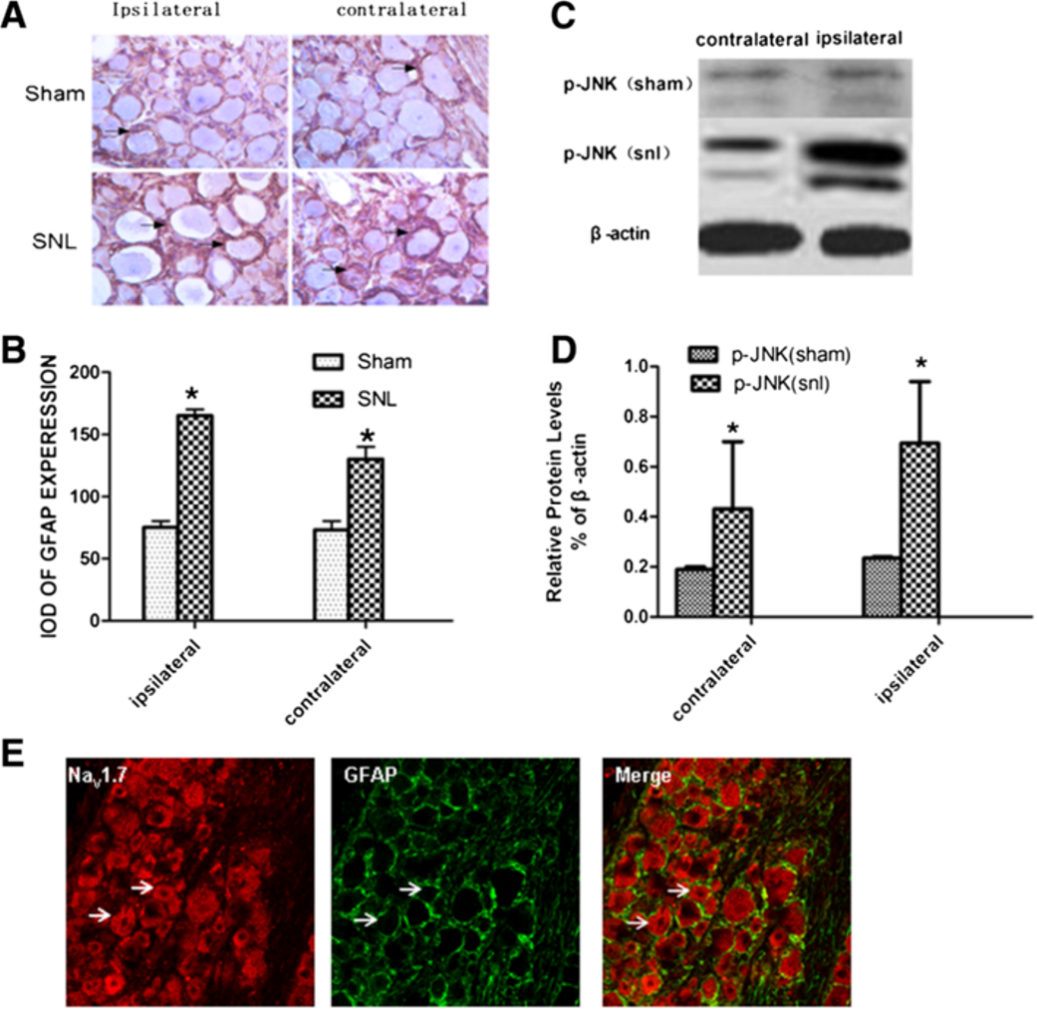
**The activated of satellite cell of bilateral ganglion. A**: GFAP expression by immunohistochemistry staining. GFAP expression was increased in the cell which around the neuron in dorsal ganglion of bilateral sides at 7 d, pointed by arrow. **B**: the statistical graph of A, *p < 0.05 vs sham group. (n = 3) **C**: western-blot showed pJNK protein was increased in dorsal ganglion of bilateral sides at 7 d, **D**: the statistical graph of E,*p < 0.05 vs sham group (n = 3). **E**: double-labelling immunofluorescence showed GFAP positive cell are around the SCN9A positive cell.

Following nerve injury, we observed a marked increase in the number of GFAP and pJNK. GFAP positive cells were found throughout the DRG and were frequently in close proximity to neurons. Interestingly, we found GFAP positive cells increased in DRG not only on the side of the nerve injury, but also on the contralateral side (Figure 3A). The integral optical density (IOD) of GFAP increased from75 ± 7.071 to 165 ± 7.071in the ipsilateral side. The IOD of GFAP increased from 73 ± 9.899 to 130 ± 14.1421in the contralateral side (*P < 0.05). Compared with the sham group, western blot revealed significant increases in pJNK in the contralateral side. (Figure 3C). The ratio to the sham group is up to 0.571 ± 0.070 from 0.191 ± 0.041 at 7 days post-SNL (*P < 0.05). Next we used anti-GFAP and anti-Nav1.7 as primary antibodies andTIFC as a secondary antibody to show satellite cells (green) of DRG and TRITC as a secondary antibody to show NaV1.7-positive cells (red). Immunofluorescence showed co-localization of GFAP and Nav1.7 in the cell (Figure 3E).

### Reversal of bilateral mechanical and thermal pain hypersensitivity by intrathecal injection of FC

**Figure 4:**
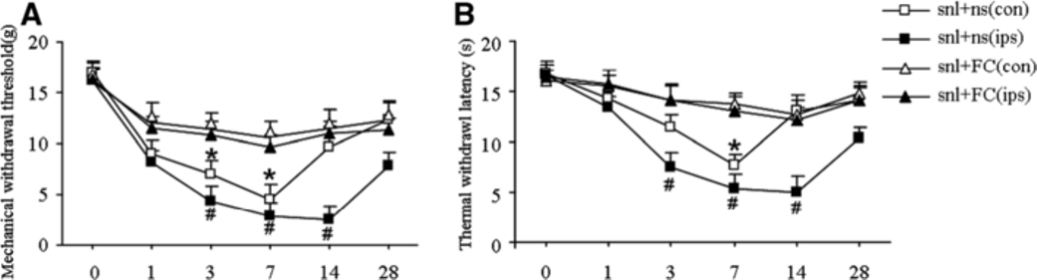
**Behavioral changes after separately intrathecal injection FC and 0.9% NS of SNL model, red arrow is sign of intrathecal injection. A**: changes of mechanical threshold of bilateral side paws: After injection FC, ipsilateral paw of SNL model showed their mechanical threshold were up, # P < 0.05 vs ipsilateral side paw of SNL + NS group. After injection FC,contralateral side paw of SNL model showed their mechanical threshold were up, *P < 0.05 vs contralateral side paw of SNL + NS group. **B**: changes of thermal threshold of bilateral side paws, after injection FC, ipsilateral paw of SNL model showed their thermal threshold were up # P < 0.05 vs ipsilateral paw of SNL + NS group. After injection FC,contralateral paw of SNL model showed their thermal threshold were up *P < 0.05 vs contralateral paw of SNL + NS group n = 6.

To further determine whether activation of satellite cells of DRG can effect the mechanical allodynia and thermal hyperalgesia, we injected FC intrathecally (i.t.) on post-SNL day 1–7 and tested mechanical and heat sensitivity after the injection. Our study showed that FC reversed mechanical allodynia in not only the ipsilateral but also the contralateral paw (P < 0.05, vs. sham groups) (Figure 4).

### The decreased GFAP in satellite cells and Nav1.7 in neurons of bilateral DRG by intrathecal injection of FC

**Figure 5:**
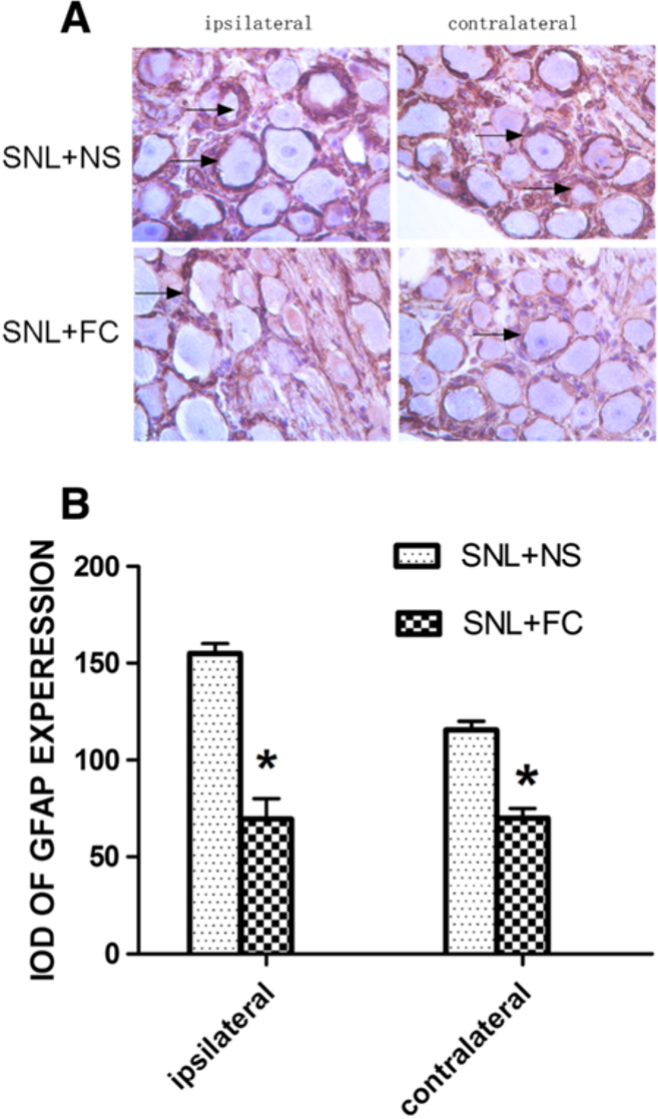
**GFAP expression in bilateral sides DRG after intrathecal injection of SNL rats at 7 d. A**: GFAP Immunohistochemistry show positive staining of satellites cell aroud neurons. **B**: average integral optical density of GFAP immunohistochemical positive area, *P < 0.05 vs SNL + NS group n = 5.

**Figure 6:**
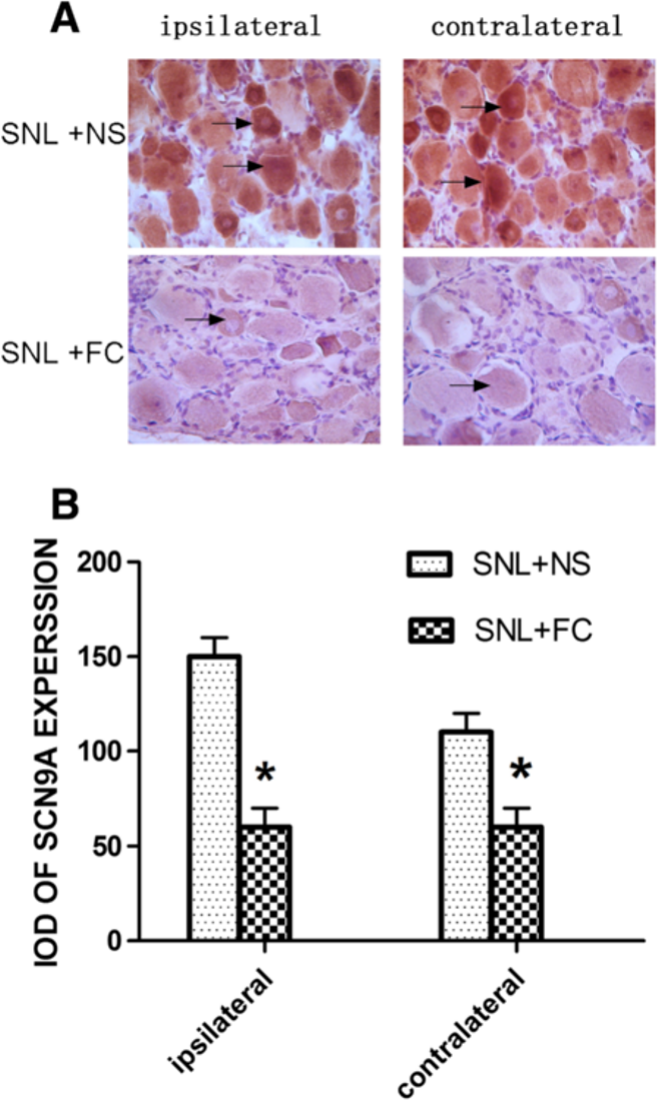
**Nava1.7 expression in bilateral sides DRG after intrathecal injection of SNL rats at 7 d. A**: Nava1.7 Immunohistochemistry show positive staining of neurons. **B**: average integral optical density of Nava1.7 immunohistochemical positive area, *P < 0.05 vs SNL + NS group n = 5.

The above data showed changes in behavior after intrathecal injection of FC. To further determine if the primary sensory neuronsdisplay changes at the cellular level, we used Immunohistochemistry to reveal changes in the expression of Nav1.7 in neurons and the expression of GFAP in satellite cells of injured L5 DRG (Figures 5 and 6). Compared with the sham groups, Immunohistochemistry revealed GFAP and Nav1.7 protein in injured L5 DRG was significantly reduced especially at 7 days post-SNL. The IOD of GFAP decreased from 155 ± 7.071 to 69.5 ± 14.849 (ipsilateral), and from 115.5 ± 6.364 to 70 ± 7.071. (*P* < 0.05). Compared with the sham groups, Immunohistochemistry revealed Nav1.7 protein in injured L5 DRG was significantly decreased especially at 7 days post-SNL. The IOD of GFAP decreased from 151 ± 15.556 to 62.5 ± 15.556 in the ipsilateral side, and from 109 ± 14.849 to 61.5 ± 4.950 in the contralateral side (*P* < 0.05).

## Discussion

After unilateral nerve injury, pain and increased pain sensitivity are evident on both sides of the body. This phenomenon is known as mirror image pain(MIP) [14]. Although not all unilateral nerve injury in clinical settings will appear as mirror pain, the contralateral side often displays hyperalgesia. So in our experiment ,we did SNL model to detected both mechanical and thermal hyperalgesia. We found that unilateral nerve injury exhibited noticeable bilateral pain. Compared with the sham group, both the ipsilateral and the contralateral paw pain thresholds for thermal and mechanical hyperalgesia were significantly increased following SNL (Figure 1). We also did immunohistochemistry and western-blot to detect the expression of Nav1.7. We found compared with the sham group, Nav1.7 [20] abnormally highly expressed in both bilateral DRG (Figure 2). Because of Nav1.7 expression is a symbol of neuron excited, high expression of Nav1.7 protein [21] in the contralateral side may explain the increased excitability of the mirror side neurons, that is consistent with the development of mirror pain. How did the contralateral side neuron excited after unilateral nerve injury? A number of studies show that glial cells can profoundly affect the genesis and/or maintenance of pain, based on this background, we focus on the glial cells of the peripheral nervous system: Satellite glial cells (SGCs). SGCs are the glial cells in DRG and are similar to astrocytes and microglia. When SGCs become activated, they may proliferate and release substances that act as messengers to excite DRG neuron. SGCs activation occurs after nerve injury or inflammation. [15, 16] In this experiment, immunohistochemistry showed SGCs are arranged in a layer, around the neurons. After unilateral nerve injury, both side SGCs were actived, we can see from (Figure 3) that GFAP and pJNK [17, 18] were increased in both side DRG. So we imagined if the SGCs involved in the mechanism of MIP. We used FC to intrathecal injection, after injection we can see from (Figure 4) that the changes in SGCs can be inhibited by intrathecal injection of FC. And then after the injection we tested mechanical and thermal sensitivity, our study showed that FC reversed mechanical allodynia not only the ipsilateral but also the contralateral paw. Regardless of whether this is the explanation,the significance of our results is that an alteration of the activation of SGCs can have significant behavioral consequences. So we can draw a conclusion from this study that after SNL, SGCs become activated [17, 18] and may release some substances ,these substances maybe the important mediators of chronic pain, as well as this experiment showed that the enhanced GFAP expression in SGCs in bilateral DRG following nerve injury. How the signal passed from the ipsilatral side to the contralateral side still remains to be answered? the answer may lie in the following two mechanisms: after neuronal injury, the pain signal will quickly reach the contralateral side SGCs from the ipsilateral side [11, 19]. But the limitation is we still need to do more about the underlying mechanisms. SGCs activation will enhance primary neuron excitability in the form of a paracrine release in the contralateral primary neurons, thus, the contralateral pain sensitivity will increase.

Because Nav1.7 increase is the symbol of neuron excited, our results showed that Nav1.7 expression increase as well as SGC activation bilaterally after unilateral nerve injury. And then after intrathecal injection of FC, Nav1.7 expression significantly decreased in the bilateral sides. So this mean inhibition of DGC activation in DRG also suppressed the excitability of neurons and pain sensitivity, these finding suggest that SGCs play an important role in the mirror pain mechanism. Based on the above studies we can draw the following conclusion: After unilateral peripheral nerve injury, SGCs become activated, leading to an increase in the expression of Nav1.7 channels in bilateral DRG, thus producing mirror pain.

### Ethics statement

This study was submitted to, and approved by, the ZhengZhou university institutional ethics committee.

## Conclusions

In conclusion, we found that SNL rats can induce MIP. Unilateral nerve injury can lead to SGCs activation and neuronal increased expression Nav1.7 in both sides of peripheral ganglia, Which may be the glial cells signal transduction between the same spinal segment. Intrathecal injection fluorocitrate can inhibit the activation of glial cells in spinal cord and DRG and reduce MIP.
